# Combined Lanreotide Autogel and Temozolomide Treatment of Progressive Pancreatic and Intestinal Neuroendocrine Tumors: The Phase II SONNET Study

**DOI:** 10.1093/oncolo/oyad325

**Published:** 2024-01-11

**Authors:** Marianne Pavel, Harald Lahner, Dieter Hörsch, Anja Rinke, Timm Denecke, Arend Koch, Benjamin Regnault, Dorit Helbig, Philipp Hoffmanns, Markus Raderer

**Affiliations:** Department of Hepatology and Gastroenterology, Charité-Universitätsmedizin Berlin, Corporate Member of Freie Universität Berlin, Humboldt-Universität zu Berlin, and Berlin Institute of Health (BIH), Berlin, Germany; Department of Medicine 1, Friedrich Alexander University Erlangen-Nuernberg, University Hospital Erlangen, Erlangen, Germany; Department of Endocrinology and Metabolism, University Hospital Essen, Essen, Germany; Department of Gastroenterology/Endocrinology, Zentralklinik Bad Berka, Bad Berka, Germany; Department of Gastroenterology, University Hospital Gießen and Marburg, Marburg and Philipps University Marburg, Germany; Department of Radiology, Campus Virchow-Klinikum, Charité – Universitätsmedizin Berlin, Berlin, Germany; Department of Diagnostic and Interventional Radiology, University Medical Center Leipzig, Leipzig, Germany; Department of Neuropathology, Charité-Universitätsmedizin Berlin, Corporate member of Freie Universität Berlin, Humboldt-Universität zu Berlin, and Berlin Institute of Health (BIH), Berlin, Germany; Ipsen Pharma, Boulogne-Billancourt, France; Ipsen Pharma GmbH, München, Germany; Ipsen Pharma GmbH, München, Germany; Medical University Vienna, Internal Medicine I, Division of Oncology, Vienna, Austria

**Keywords:** neuroendocrine tumors, gastrointestinal neoplasms, O(6)-methylguanine-DNA methyltransferase, temozolomide, lanreotide, receptors, somatostatin

## Abstract

**Background:**

In advanced neuroendocrine tumors (NET), antiproliferative treatment options beyond somatostatin analogs remain limited. Temozolomide (TMZ) has shown efficacy in NET alone or combined with other drugs.

**Materials and Methods:**

SONNET (NCT02231762) was an open, multicenter, prospective, phase II study to evaluate lanreotide autogel 120 mg (LAN) plus TMZ in patients with progressive advanced/metastatic grade 1/2 gastroenteropancreatic (GEP) NET or of unknown primary. Patients could be enrolled at first-line or higher therapy line. The primary endpoint was disease control rate ([DCR], rate of stable disease [SD], partial [PR], and complete response [CR]) at 6 months of LAN and TMZ. Patients with nonfunctioning (NF) NET without progression at 6 months were randomized to 6-month LAN maintenance or watch and wait, patients with functioning (F)-NET with clinical benefit (PR, SD) continued on LAN.

**Results:**

Fifty-seven patients were recruited. The majority of patients received the study drug at second or higher treatment line and had an NET G2. DCR at 6 months LAN and TMZ was 73.5%. After 6 months of further LAN maintenance, 54.5% of patients with F-NET and 71.4% with NF-NET had SD or PR vs 41.7% with NF-NET on observation only. LAN and TMZ were effective in all subgroups analyzed. At 12 months of follow-up, median progression-free survival was 11.1 months. Median serum chromogranin A decreased except in NF-NET on observation. O6-methylguanine DNA methyltransferase promoter methylation appeared to better reflect TMZ response than loss of gene expression. During combination therapy, the most frequent treatment-emergent adverse events grade 3/4 reported were nausea (14%), thrombocytopenia (12.3%), and neutropenia (8.8%). Four deaths were reported resulting from severe adverse events not considered related to study medication.

**Conclusions:**

LAN plus TMZ is a treatment option for patients with progressive GEP-NET with more aggressive biological profile showing a manageable safety profile.

**Implications for Practice**

Neuroendocrine tumors of the gastrointestinal tract or pancreas are neoplasms that originate from cells of the diffuse neuroendocrine cell system. Drugs that control tumor growth and/or inhibit hormone release are a mainstay of management of neuroendocrine tumors. This study investigated the combined treatment with lanreotide, a drug derived from the naturally occurring inhibitory hormone somatostatin, and the chemotherapeutic agent temozolomide. The results show that lanreotide plus temozolomide provided clinical benefit in patients who have failed on other therapies or for whom other therapies are unsuitable. The combined treatment with addition of lanreotide to temozolomide does not seem to result in more adverse events than treatment with temozolomide alone. Less than 10% of the patients discontinued the trial due to side effects related to the study drug.

## Background

Neuroendocrine tumors (NET) are a heterogeneous group of rare malignancies most often localized in the gastrointestinal tract, lung, and pancreas. Reported incidence is up to 7/100 000.^1,2^ Prognosis depends on primary site, presence of metastatic disease, tumor grade and stage at diagnosis as well as hormonal secretion. At presentation, most patients have unresectable disease due to local extension or metastases.^1^ Strategies of gastroenteropancreatic (GEP) NET treatment include tumor removal, control of tumor growth and symptoms as well as maintenance of quality of life (QoL).^3^ Metastasized well-differentiated low-grade NET are usually controlled by antisecretory and antiproliferative treatments such as somatostatin analogs or targeted therapies, as well as by local treatments and peptide receptor radionuclide therapy (PRRT).^4^

For somatostatin analogs, 2 placebo-controlled trials have demonstrated antiproliferative activity. The PROMID study showed improvement in time to progression in therapy-naïve midgut NET by octreotide long-acting release.^5^ In the CLARINET study,^6^ lanreotide autogel (LAN) resulted in prolonged progression-free survival (PFS) vs placebo in patients with metastatic grade 1 or 2 (Ki-67 ≤10%) somatostatin receptor (SSTR)-positive enteropancreatic nonfunctioning (NF)-NET with prior stable disease (SD). Tumor stabilization and PFS >12 months after LAN 120 mg was also demonstrated in a Spanish open-label phase II study in patients with progressive NET.^7^

In Europe, LAN 120 mg/4 weeks is currently licensed for the treatment of grade 1 and a subset of grade 2 (Ki-67 index ≤10%) GEP-NET of midgut, pancreatic, or unknown origin where hindgut sites of origin have been excluded, in patients with unresectable locally advanced or metastatic disease. In addition, it is indicated for the treatment of symptoms associated with NET (particularly carcinoid tumors).^8^ In the US, it is approved for the treatment of patients with unresectable, well- or moderately differentiated, locally advanced, or metastatic GEP-NET to improve PFS, and, in addition, for the treatment of patients with carcinoid syndrome.^9^

In advanced NET, however, antiproliferative treatment options beyond somatostatin analogs remain limited. According to current guidelines,^4,10^ PRRT is a therapeutic option in progressive SSTR-positive NET with homogenous SSTR expression. PRRT may be recommended in midgut NET as a second-line therapy after failure of somatostatin analogs in patients fulfilling the minimal general requirements for PRRT^11^ and may be considered as well at further therapy lines and in NET from other sites than midgut.^4,10^ The use of systemic chemotherapy is usually restricted to poorly differentiated neuroendocrine carcinoma, or NET with higher proliferative activity, particularly if of pancreatic or pulmonary origin. Streptozotocin-based chemotherapy is one of the treatment options approved in pancreatic NET but is not widely available in all countries.^12^ The alkylating agent temozolomide (TMZ) is an alternative option, and showed promising efficacy in a retrospective study in pancreatic NET and in a limited number of small studies in mixed patient populations, both as monotherapy^13^ or in combination with bevacizumab,^14^ thalidomide,^15^ everolimus,^16^ or capecitabine.^17-20^ A prospective phase II study in pancreatic NET recently showed similar overall response rates (ORR) for TMZ vs capecitabine and TMZ; however, the combination regimen was associated with more durable PFS—but also included more NET G1 with a more favorable prognosis.^21^ While some studies found superior response to TMZ in association with promoter methylation or loss of gene expression of the DNA repair enzyme O6-methylguanine DNA methyltransferase (MGMT),^22,23^ other studies failed to show this relationship. Altogether, contradictory results exist concerning MGMT status as a predictive molecular marker in patients with NET treated with alkylating agents, and data from a prospective evaluation are missing so far.^24,25^

The aim of this study was to prospectively evaluate the efficacy and safety of combined LAN and TMZ as an alternative treatment option in patients with progressive advanced/metastatic grade 1 or 2 GEP-NET or NET (ie, cancer) of unknown primary origin (NET-CUP).

## Materials and Methods

### Patients

Patients ≥18 years of age diagnosed with advanced unresectable or metastatic differentiated GEP-NET grade 1 or 2 (Ki-67 index ≤20%) or NET-CUP confirmed by pathological/histological assessment and progressive disease (PD) within 12 months before inclusion according to Response Evaluation Criteria In Solid Tumors (RECIST) version 1.1^26^ by computed tomography (CT) or magnetic resonance imaging (MRI) and a World Health Organization (WHO) performance score 0-2 could be included. Resectability of the tumor lesions was assessed in interdisciplinary tumor boards of participating centers based on distribution and exact localization of liver lesions following current guidelines recommendations.^4,10^ Patients had to have a positive SSTR status as assessed by SSTR scintigraphy (including single photon emission tomography (SPECT or SPECT/CT) or SSTR positron emission tomography (PET)/CT) within 12 months before screening as confirmed by an independent central read. Absolute neutrophil count had to be ≥1.5 × 10^9^/L prior to TMZ administration (thrombocyte count, ≥100 × 10^9^/L). Main exclusion criteria were insulinoma or multiple endocrine neoplasia, glycated hemoglobin >8.5%, therapy with sunitinib, everolimus, or interferon-α within 4 weeks prior to screening, TMZ at any time; PRRT (≤3 months before inclusion), local ablative therapies (≤6 months), irradiation of target lesions (≤12 months), and major surgery (≤3 months).

### Study Design

**Figure 1.**
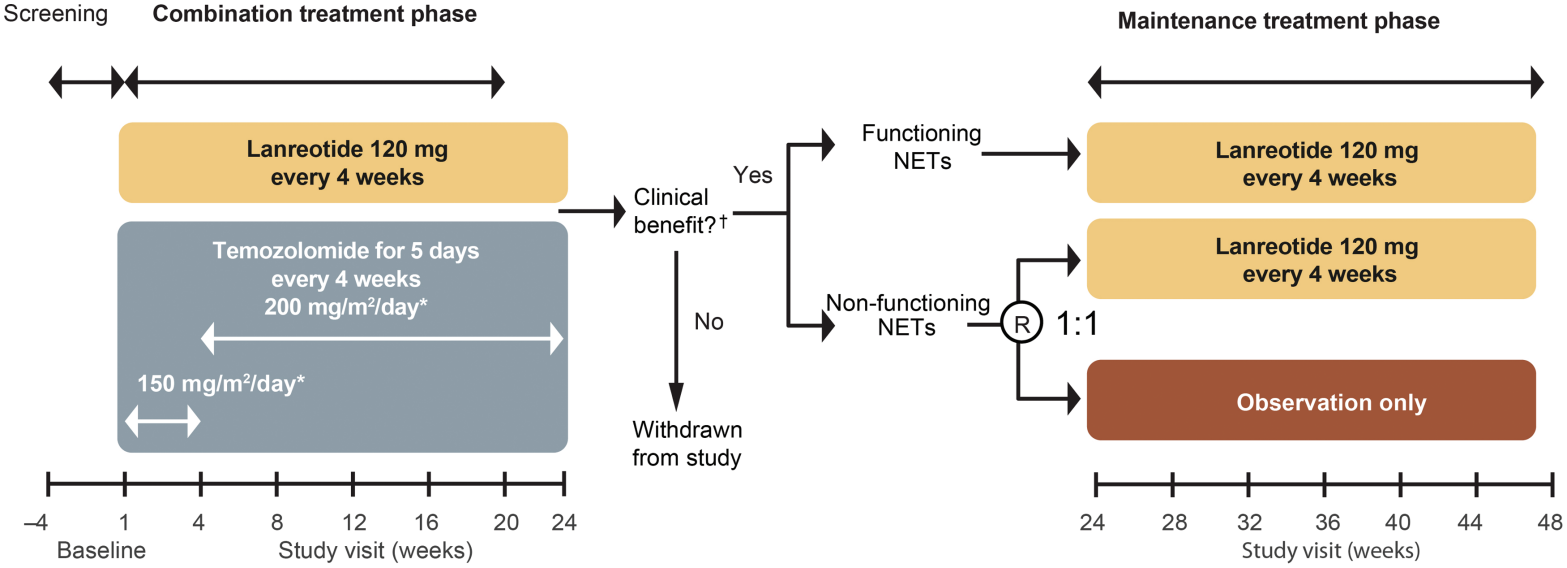
Study design. *Temozolomide dose increased during the combination phase, ^†^defined as a complete or partial response or stable disease assessed centrally using RECIST v1.1. Abbreviations: NET, neuroendocrine tumor; R, randomization.

SONNET was an open-label, prospective, multicenter, noncomparative phase II study (EudraCT: 2013-001697-17; Clinical Trials.gov: NCT02231762). All patients received treatment with LAN 120 mg/4 weeks with TMZ for the first 6 months (Figure 1; combination treatment phase). TMZ was administered for 5 consecutive days every 28 days at 150 mg/m^2^/day for 1 month, then increased to 200 mg/m^2^/day depending on the safety laboratory values. In case of clinical benefit of combination treatment, defined as either tumor remission or SD according to RECIST 1.1, patients with functioning (F)-NET received LAN 120 mg/4 weeks for another 6 months as maintenance treatment; patients with NF-NET and clinical benefit after 6 months were randomized to LAN 120 mg/4 weeks or no treatment following a watch-and-wait concept (Fig. 1; maintenance treatment phase).

The primary endpoint was disease control rate (DCR) after 6 months of combination treatment. DCR was imaging based and defined as a combination of the RECIST 1.1 categories “complete response” (CR), “partial response” (PR), or “stable disease” (=nonPD). Main secondary endpoints were DCR after 12 months (LAN 120 mg maintenance or watch and wait), time to progression or death occurring within 12 months after first combination treatment (statistical estimation of PFS), biochemical response after 6 and 12 months based on increase or decrease of serum chromogranin A (CgA) levels (SD defined as <50% decrease or ≤25% increase from baseline; PD: ≥25% increase as best response; PR: decrease of ≥50%) and urine 5-hydroxyindoleacetic acid (5-HIAA) levels only in patients with F-NET (carcinoid syndrome) (response defined as 5-HIAA reduction compared to baseline, progression defined as 5-HIAA increase). DCRs were analyzed according to the following variables at baseline and categories: functional status (F-NET vs NF-NET), NET location (pancreatic, small intestinal, other), tumor grade according to the WHO 2010 classification (G1, proliferation index Ki-67 <3% and G2, Ki-67 3%-20%),^27^ and hepatic tumor load (≤25%, >25%). Additionally, DCR according to Ki-67 categories (<5%, 5%-10%, and >10%-20%) were studied. Analysis of further Ki-67 categories was based on the evidence of improved prognostic stratification from previous publications.^28-31^

Other endpoints included symptomatic response in patients with F-NET, changes of QoL questionnaire scores as well as MGMT expression and methylation, and SSTR expression in correlation with DCR.

For standardization of the imaging-based endpoints throughout the study population, CT/MRI examination protocols were harmonized with a prescribed minimal standard via the study protocol regarding imaging technique including multiphasic contrast-enhanced CT/MRI according to the European Neuroendocrine Tumor Society guidelines.^32^ As per study protocol, CT or MRI examinations were performed at baseline and every 12 weeks until the end of the study. The study was conducted in compliance with independent ethics committees/institutional review boards, the Declaration of Helsinki (Version 2013), and International Conference on Harmonisation (ICH) Good Clinical Practice (GCP) Guidelines. All patients provided written informed consent.

### Assessments

For determination of the imaging-based endpoints, the CT and MRI examinations were pseudonymized and forwarded to a central server and underwent an independent blinded read applying RECIST 1.1 by an experienced radiologist and nuclear medicine specialist (T.D., 15 years of experience with NET imaging).

Routine laboratory assessments, serum CgA, and urine 5-HIAA determinations were performed at the Spranger Laboratories, Ingolstadt, Germany. CgA was measured by an enzyme-linked immunosorbent assay (IBL International, Hamburg, Germany).

QoL was assessed by the German-validated version of the European Organization for Research and Treatment of Cancer (EORTC) Study Group’s 30-item Quality of Life Questionnaire (QLQ-C30) (subscores: global health status; physical, role, emotional, cognitive, and social functional scales; and symptom scales) and the 21-item EORTC QLQ-GINET21 questionnaire (subscores: treatment-related symptoms, weight gain, information/communication function, sexual function, endocrine symptoms, gastrointestinal symptoms, social function, disease-related worries, muscle/bone pain symptoms, and body image).

#### Immunohistochemistry

Immunohistochemical stainings were performed on a Benchmark XT autostainer (Ventana Medical Systems, Tuscon, AZ, USA) with standard antigen retrieval methods and according to standard procedures. With regard to the immunohistochemical staining with monoclonal antibodies against SSTR-2a (clone MMB1diluted at a ratio of 1:200; abcam) and 5 (clone MMB4 diluted at a ratio of 1:100; abcam), the proportion of membrane-bound positive tumor cells was evaluated using a scoring scheme (score 0: no expression, score 1: cytoplasmic expression, score 2: incomplete circumferential expression in less than 50% of tumor cells).^33^

MGMT protein expression was performed using a monoclonal MGMT antibody (clone MT23.2, diluted at a ratio of 1:50; Invitrogen). MGMT showed strong nuclear reactivity in internal controls (lymphocytes and endothelial cells). Positive MGMT staining was defined as the staining intensity of the majority of tumor cells >50% similar to that of the adjacent endothelial cells. Negative MGMT staining was defined as tumor cells with no or weak staining than that of endothelial cells. We used the same semiquantitative immunohistochemical procedure described elsewhere.^34^

#### MGMT Promoter Methylation Status in Tumor Samples

Areas of high tumor cell content (>70%) were chosen and macrodissected for further analysis. Genomic DNA was extracted from paraffin-embedded tumor samples using the Qiagen DNeasy blood and tissue DNA extraction kit according to the manufacturer’s protocol (Qiagen, Hilden, Germany). After sodium bisulfite modification, quantitative methylation analyses were performed by pyrosequencing analysis described elsewhere.^35^ The percentage of methylated alleles was calculated as the mean of the obtained methylation percentage. The cutoff value of >10% was defined to classify MGMT-methylated vs nonmethylated cases, which is commonly used and validated for routine clinical diagnostics in gliomas.^36^

#### Safety Assessments

Safety assessments included vital signs, biochemistry, and hematology laboratory values. Adverse events (AE) were reported using the National Cancer Institute Common Terminology Criteria for Adverse Events (NCI-CTCAE) classification (Version 4.0), coded using Medical Dictionary for Regulatory Activities (MedDRA) Version 18.0, and classified by MedDRA preferred term and system organ class. The presence of gallbladder stones was assessed by ultrasound at baseline and at months 12 and 24 by the investigators.

### Statistical Analysis

The single-arm study was designed to demonstrate that DCR after 6 months of combination treatment was greater than 45%, judged to be clinically meaningful. Assuming an expected proportion of subjects with a DCR after 6 months of combination treatment of 70% and 40 evaluable subjects, the study had a power of 88% to demonstrate that the proportion of subjects with DCR after 6 months was greater than 45% with a 2-sided alpha of 0.05 (equivalent to a one-sided alpha of 0.025) and using exact methods. All efficacy endpoints were analyzed in the intent-to-treat (ITT) population by means of descriptive statistical methods (mean, range [minimum, maximum], median, and interquartile range [IQR]); the primary endpoint was described along with its respective 95% CI. Kaplan-Meier estimate of PFS was constructed and median PFS was obtained along with 95% CI for all patients included in the study as well as for subgroups such as MGMT methylation and expression status. Symptomatic response was evaluated from absolute changes from baseline in the number of episodes for diarrhea and flushing per day using the mean of the last 3 days before the visit at each visit as compared to baseline. QoL subscores were described with changes from baseline in QLQ-C30 and GINET21 scores at each visit. Descriptive analyses of subgroups on F-/NF-NET, primary tumor localization, grade, and hepatic tumor burden were performed on specific efficacy tables. Statistical analysis was performed using Statistical Analysis System software version 9.4 (SAS Institute Inc., Cary, NC, USA).

## Results

### Patient Disposition

Fifty-seven of 64 screened patients with advanced NET started the combination phase and received combined treatment with LAN 120 mg/4 weeks and TMZ. Screening failures included patients who did not meet entry criteria (*n* = 5), consent withdrawal (*n* = 1), and an adverse event (*n* = 1) that occurred after informed consent and before first administration of the study medication. Thirty-seven patients completed the combination phase and had clinical benefit (Supplementary Fig. S1). Of these, 11 had F-NET and received LAN 120 mg/4 weeks and 26 had NF-NET and were randomized equally to receive either LAN 120 mg (14 patients) or no treatment (12 patients). Seventeen patients treated with LAN 120 mg (F-NET, *n* = 8; NF-NET, *n* = 9) and 7 nontreated patients (watch and wait) completed the maintenance phase. During the combination phase, the proportion of patients who were assessed for the primary efficacy parameter (86.0%) and who discontinued treatment from this phase (35.1%) was similar to the proportions in the maintenance phase (86.2% and 35.1%, respectively). During the combination phase treatment discontinuations (*n* = 20) were mainly due to AE (9 [45.0%] patients) and disease progression (6 [30.0%] patients); discontinuations during the maintenance phase (*n* = 13) were mainly due to disease progression (10 [76.9%] patients) and AE (3 [23.1%] patients) (Supplementary Fig. S1).

### Patient and Tumor Characteristics

Median age of the enrolled patients was 65.0 years, most of them were male (Table 1). Median time from diagnosis was 24.5 months. Primary tumor location was mainly small intestinal (43.9%) followed by pancreatic (35.1%) and colorectal (7.0%), about one-third were functionally active (36.8%). The majority (84.2%) of patients presented with grade 2 disease, 86% with distant metastases. In more than half of the patients, hepatic tumor load exceeded 10% (Table 1).

**Table 1.** Baseline characteristics of the enrolled patients.

Variable	Value
Median age, years (range)	65.0 (39-82)
Gender, *n* (%) (*N* = 57)	
Male	33 (57.9)
Female	24 (42.1)
Median time since NET diagnosis, months (range)	24.5 (1.0-171.8)
Primary tumor location, *n* (%) (*N* = 57)	
Pancreatic	20 (35.1)
Small intestinal	25 (43.9)
Colorectal	4 (7.0)
Other	2 (3.5)
Unknown	6 (10.5)
NET tumor grade according to WHO 2010 classification,^27^*n*/*N* (%)	
Grade 1 (Ki-67 <3%)	9/57 (15.8)
Pancreatic NET	1/20 (5.0)
Small intestinal NET	5/25 (20.0)
Other	3/12 (25.0)
Grade 2 (Ki-67 3%-20%)	48/57 (84.2)
Pancreatic NET	19/20 (95.0)
Small intestinal NET	20/25 (80.0)
Other	9/12 (75.0)
Distant metastasis, *n* (%)	49 (86.0)
Proliferation index	
Ki-67 categories, *n* (%) (*N* = 57)	
<5%	17 (29.8)
5%-10%	26 (45.6)
>10%-20%	14 (24.6)
Functional activity, *n* (%) (*N* = 57)	
Functioning	21 (36.8)
Pancreatic NET	3 (5.3)
Small intestinal NET	14 (24.6)
Other NET	4 (7.0)
Nonfunctioning	36 (63.2)
Pancreatic NET	17 (29.8)
Small intestinal NET	11 (19.3)
Other NET	8 (14.0)
Hepatic tumor load, *n* (%) (*N* = 57)	
0%	2 (3.5)
>0%-≤10%	24 (42.1)
>10%-≤25%	20 (35.1)
>25%-≤50%	4 (7.0)
>50%	6 (10.5)
Unknown	1 (1.8)
Prior surgical procedures, *n* (%) (*N* = 57)	
Patients with surgical procedure	37 (64.9)
Partial pancreatectomy	8 (14.0)
Hemicolectomy	6 (10.5)
Ileum segment resection/ileocecal resection	6 (10.5)
Cancer surgery	5 (8.8)
Lymphadenectomy	5 (8.8)
Splenectomy	5 (8.8)
Cholecystectomy	4 (7.0)
Hepatectomy	4 (7.0)
Ileal operation	4 (7.0)
Small intestinal resection	4 (7.0)
Explorative laparotomy	2 (3.5)
Hepatic embolization	2 (3.5)
Intestinal anastomosis	2 (3.5)
Resection of rectum	2 (3.5)
Tumor excision	2 (3.5)
Appendectomy	1 (1.8)
Prior medical treatment, *n* (%) (*N* = 57)	
Patients with any prior therapy	47 (82.5)
Somatostatin analogs	34 (59.6)
Everolimus	10 (17.5)
Streptozotocin + 5-fluorouracil	9 (15.8)
Doxorubicin	2 (3.5)
Cisplatin + etoposide	2 (3.5)
Sunitinib	2 (3.5)
Gemcitabine	1 (1.8)
Interferon-α	1 (1.8)
Prior PRRT, *n*/*N* (%)	29 (50.9%)
Tissue SSTR-2A expression, *n*/*N* (%)	
None (score 0)	0/38 (0.0)
Cytoplasmatic expression (score 1)	0/38 (0.0)
Focal expression (score 2)	14/38 (36.8)
Complete circumferential membrane expression (score 3)	24/38 (63.2)
Tissue SSTR-5 expression, *n*/*N* (%)	
None (score 0)	25/38 (65.8)
Cytoplasmatic expression (score 1)	2/38 (5.3)
Focal expression (score 2)	11/38 (28.9)
Complete circumferential membrane expression (score 3)	0 (0.0)

Abbreviations: PRRT, peptide receptor radionuclide therapy; SSTR, somatostatin receptor; NET, neuroendocrine tumor; WHO, World Health Organization.

Prior surgical procedures for GEP-NET were reported in 37 (64.9%) patients (Table 1). Procedures included (multiple procedures in the same patient possible) partial pancreatectomy in 8 (14.0%) patients, hemicolectomy in 6 (10.5%) patients, ileum segment resection/ileocecal resection in 6 (10.5%) patients, cancer surgery in 5 (8.8%) patients, lymphadenectomy in 5 (8.8%) patients, splenectomy in 5 (8.8%) patients, cholecystectomy in 4 (7.0%) patients, hepatectomy in 4 (7.0%) patients, ileal operation in 4 (7.0%) patients, small intestinal resection in 4 (7.0%) patients, explorative laparotomy in 2 (3.5%) patients, hepatic embolization in 2 (3.5%) patients, intestinal anastomosis in 2 (3.5%) patients, resection of rectum in 2 (3.5%) patients, tumor excision in 2 (3.5%) patients, and appendectomy in 1 (1.8%) patient (Table 1). Forty-seven (82.5%) patients had at least one prior systemic therapy for NET, of whom 34 (59.6%) had somatostatin analogs (octreotide or lanreotide), 12 (21.1%) patients tyrosine kinase inhibitors (TKI), 29 (50.9%) patients had prior PRRT (Table 1). Patients had received a mean of 1.6 (range, 0-4) prior treatment lines.

All examined tumor samples (*n* = 38) showed a strong incomplete (score 2; 36.8%) or complete (score 3; 63.2%) membrane-bound expression of the SSTR-2a. The SSTR-5 receptor was not expressed in about two-thirds of the cases (score 0) and was incompletely expressed (score 2) in about one-third of the cases (Table 1).

### Disease Control and Progression

**Figure 2.**
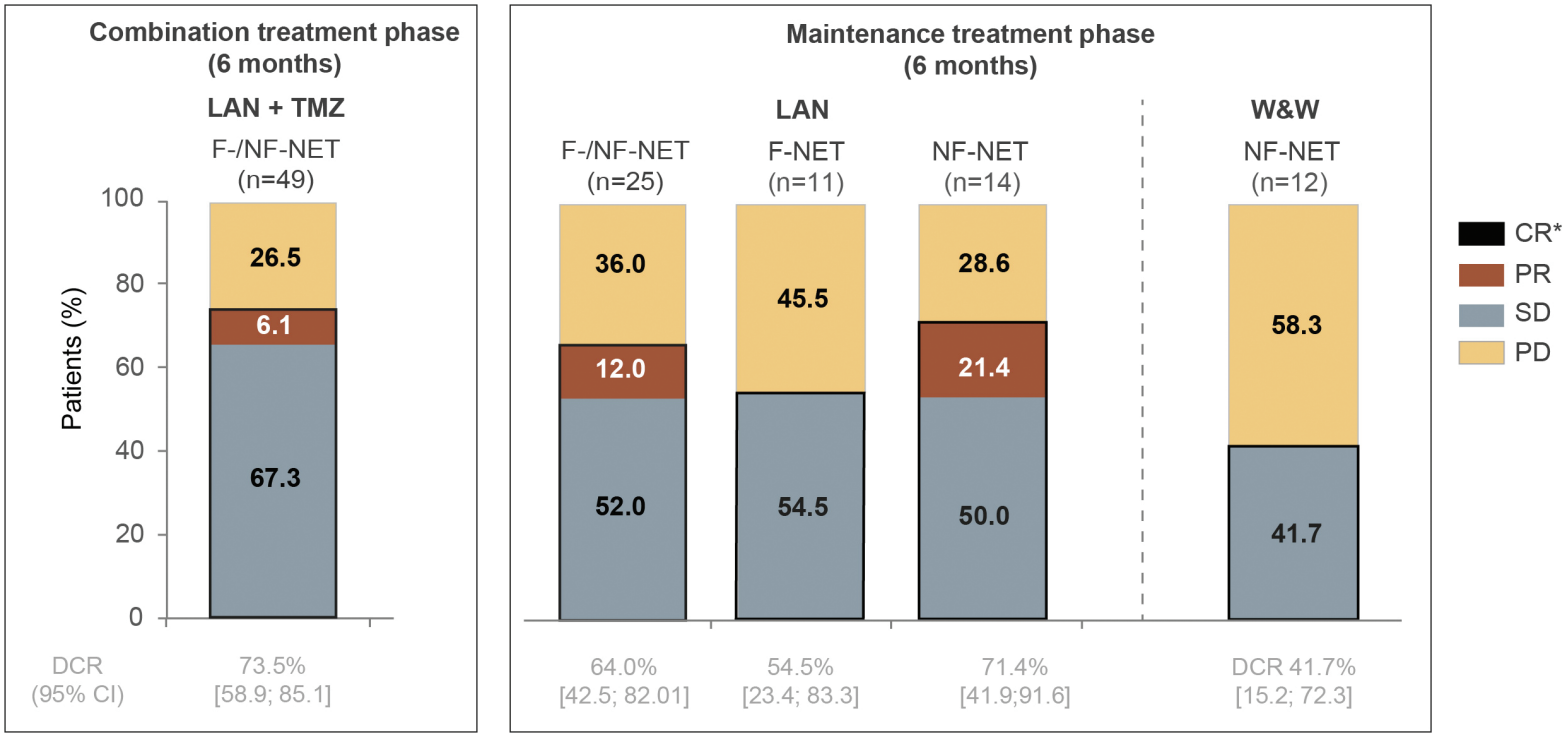
DCR at 6 months of combination phase treatment and at further 6 months of maintenance phase treatment of NET patients. The first 3 columns of the right figure panel show overall response to LAN during maintenance treatment in patients with F- and NF-NET combined as well as separately. *No CR was observed during the study. Abbreviations: F, functional; CR, complete response; DCR, disease control rate (=CR + PR + SD); LAN, lanreotide autogel; NET, neuroendocrine tumor; NF, nonfunctioning; PD, progressive disease; PR, partial response; SD, stable disease; TMZ, temozolomide; W&W, watch and wait.

DCR after 6 months of combination treatment was chosen as primary endpoint since it well reflects the clinical benefit of the patients. In our study, 36 from 49 (73.5%) patients had PR (*n* = 3; 6.1%) or SD (*n* = 33; 67.3%) at 6 months of treatment with LAN and TMZ (Fig. 2). After further 6 months of maintenance therapy or observation, 6 (54.5%) LAN-treated patients with F-NET, 10 (71.4%) LAN-treated patients with NF-NET, and 5 (41.7%) patients with NF-NET on observation showed disease control (Fig. 2). Summarized DCR for all patients with F-NET and NF-NET treated with LAN during the maintenance treatment phase (*n* = 25) was 64% (Fig. 2).

At the end of the combination phase, DCRs were numerically similar between patients with F-and NF-NET (*n* = 12; 70.6% and *n* = 24; 75.0%, respectively; Table 2). Higher DCR was observed in the following subgroups: NF-NET, primary tumor in small intestinal or other locations, grade 1, and Ki-67 <5% (Supplementary Table S1).

**Table 2.** DCR after 6 months by MGMT status—combination phase (*n* = 41)^a^.

Response according to MGMT methylation		
	Methylated (*n* = 11)	Not methylated (*n* = 15)
PD, *n* (%)	1 (9.1)	4 (26.7)
SD, *n* (%)	10 (90.9)	9 (60.0)
PR, *n* (%)	0.0	2 (3.3)
CR, *n* (%)	0.0	0.0
DCR (SD + PR + CR), *n* (%)	10 (90.9)	11 (73.3)

Response according to MGMT expression		
	Expression (*n* = 11)	No expression (*n* = 21)
PD, *n* (%)	1 (9.1)	6 (28.6)
SD, *n* (%)	9 (81.8)	14 (66.7)
PR, *n* (%)	1 (9.1)	1 (4.8)
CR, *n* (%)	0.0	0.0
DCR (SD + PR + CR), *n* (%)	10 (90.9)	15 (71.4)

^a^8 patients of the ITT population (*n* = 49) not included due to missing imaging data and/or missing tissue for primary endpoint analysis at 6 months; MGMT methylation or expression were not evaluable in 15 and 9 patients despite available tissue samples, respectively.

Abbreviations: CR, complete response; DCR, disease control rate; ITT, intent-to-treat; MGMT, O6-methylguanine DNA methyltransferase; PD, progressive disease; PR partial response; SD, stable disease.

At the end of the combination phase, PR was observed in 3 (9.4%) patients with NF-NET, 2 of them with a pancreatic primary tumor. At the end of the maintenance phase, PR was observed in 3 (21.4%) LAN-treated patients with NF-NET, all of them with pancreatic primary tumors. Six (16.2%) patients had PD and no patient had a CR in any treatment phase during the study. Overall median estimated PFS was 11.1 months (95% CI, 8.3—not calculated [NC]) across all patients, comparable to the subgroups of patients with pancreatic or small intestinal NET (Supplementary Fig. S2).

### MGMT Status and Tumor Response (DCR)

Tissue samples from 42 tumors were sent in for pathological examination with imaging data at 6 months for primary endpoint evaluation being available for 41 patients. Twenty-seven tissue samples were evaluable for *MGMT* promoter methylation. In 11 (40.7%) of these tumors, the *MGMT* promoter was methylated and in 16 (59.3%), it was not methylated. MGMT protein expression was evaluable in 33 tissue samples. MGMT was expressed in 12 (36.4%) tumors, whereas 21 (63.6%) tumor samples did not show nuclear MGMT reactivity. In order to study whether *MGMT* methylation correlates with a loss of MGMT protein expression, the cases were evaluated in which both *MGMT* methylation status and MGMT protein expression were present. Here, 63% of the cases with *MGMT* methylation concurrently showed a loss of MGMT protein expression, whereas in 37% this correlation was not demonstrated.

Analysis of DCR according to MGMT status was performed in patients of the ITT population, ie, for whom both, imaging results for evaluation of the primary endpoint of DCR at 6 months of treatment and sufficient tissue for molecular analysis were available (*n* = 41; Table 2). Eight patients of the ITT population were not included in this analysis due to missing imaging data at 6 months of treatment and/or lack of evaluable tissue. Ten from 11 (90.9%) patients with methylated gene promoter had controlled disease (all SD) and 1 patient (9.1%) experienced disease progression (Table 2). From 15 patients lacking *MGMT* promoter methylation, 11 (73.3%) had controlled disease (60.0% SD, 6.7% PR) and 4 (26.7%) progressed. From 11 patients with MGMT expression, 10 (90.9%) had disease control (81.8% SD, 9.1% PR) and 1 (9.1%) patient experienced disease progression. Expression was lost in 21 patients. Fifteen of these (71.4%) had controlled disease (66.7% SD, 4.8% PR) and 6 (28.6%) had disease progression (Table 2). No association was observed between MGMT status and PFS (not shown).

### Biochemical Response

**Figure 3.**
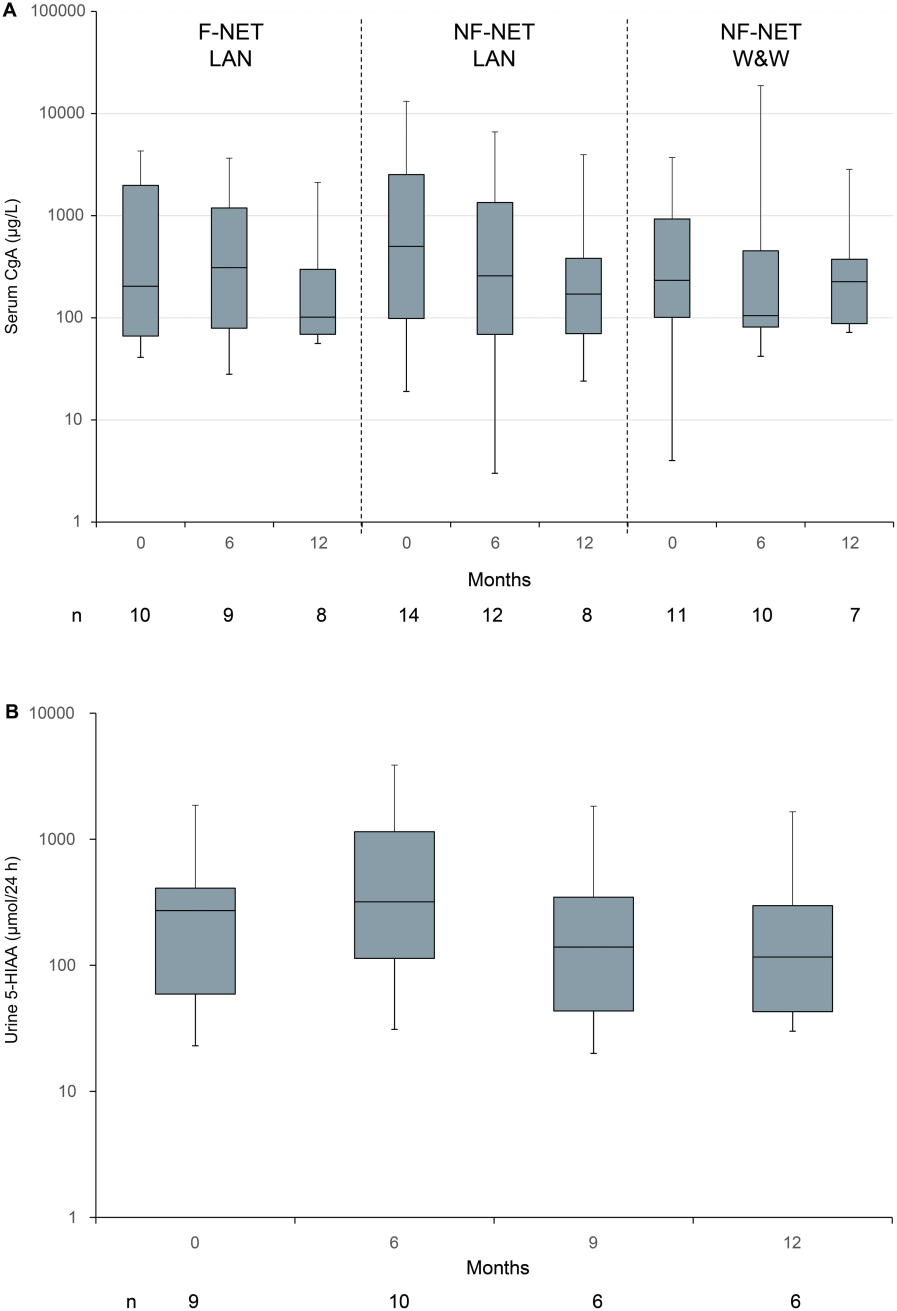
Box-Whisker plot. (**A**) Median serum CgA levels and IQR (first and third quartile) of GEP-NET patients according to subgroups of the maintenance phase; CgA normal range: <100 µg/L. (**B)** Median 5-HIAA levels and IQR (first and third quartile) of the group of LAN-treated patients with carcinoid syndrome; 5-HIAA normal range: 31.4-52.3 µmol/24 h. Changes from baseline to month 6 reflect combination phase and from months 6 to 12 maintenance phase. Abbreviations: CgA, chromogranin A; F, functioning; HIAA, hydroxyindoleacetic acid; IQR, interquartile range; LAN, lanreotide autogel; NET, neuroendocrine tumor; NF, nonfunctioning; W&W, watch and wait.

At baseline overall median serum CgA levels were 509.8 µg/L (IQR 98.5-2445.2) (*n* = 48), 34 (69.4%) patients had CgA levels ≥100 μg/L. Median CgA levels decreased to 200.6 µg/L (IQR 80.2-914.6) at the end of the combination phase (*n* = 32) and to 156.9 µg/L (IQR 73.3-374.3) at month 12 (*n* = 37) irrespective of the type of maintenance therapy. SD by CgA levels was observed in 9 patients and PR in 7 patients at the end of the combination phase. During combined treatment, serum CgA levels numerically increased in patients with F-NET from 203.7 µg/L (IQR 66.3-1978.9) to 310.3 µg/L (IQR 79.1-1189.0) at 6 months (*n* = 9) and during LAN maintenance, numerically decreased to 101.6 µg/L (IQR 69.0-298.1) at month 12 (*n* = 8). In patients with NF-NET, CgA levels decreased during combined treatment from 500.6 µg/L (98.5-2524.4) (*n* = 14) to 257.5 µg/L (68.8-1343.7) at 6 months (*n* = 12) in patients who then entered LAN maintenance and from 232.6 µg/L (IQR 101.1-927.4) (*n* = 11) to 105.0 µg/L (IQR 81.3-453.3) at month 6 (*n* = 10) in patients who had been randomized into the watch-and-wait group. At month 12, CgA levels further decreased in LAN-treated patients to 170.8 µg/mL (IQR 70.0-381.9) (*n* = 8) but returned to baseline levels in patients without LAN therapy (226.0 µg/mL [IQR 87.8-374.3], *n* = 7). Figure 3A shows serum CgA levels according to the treatment groups of the maintenance phase.

In patients with F-NET, median urine 5-HIAA levels decreased from 350.4 µmol/24 h (IQR 140.5-848.1) at baseline (*n* = 12) to 318.8 µmol/24 h (IQR 113.5-1147.5) in month 6 (*n* = 10). During the maintenance phase, median urine 5-HAA levels further decreased to 116.2 mg/24 h (IQR 42.9-297.1) in month 12 (*n* = 6). Figure 3B shows the course of 5-HIAA levels from baseline in patients with F-NET who continued to receive LAN during the maintenance phase.

### Symptoms and Quality of Life

**Figure 4.**
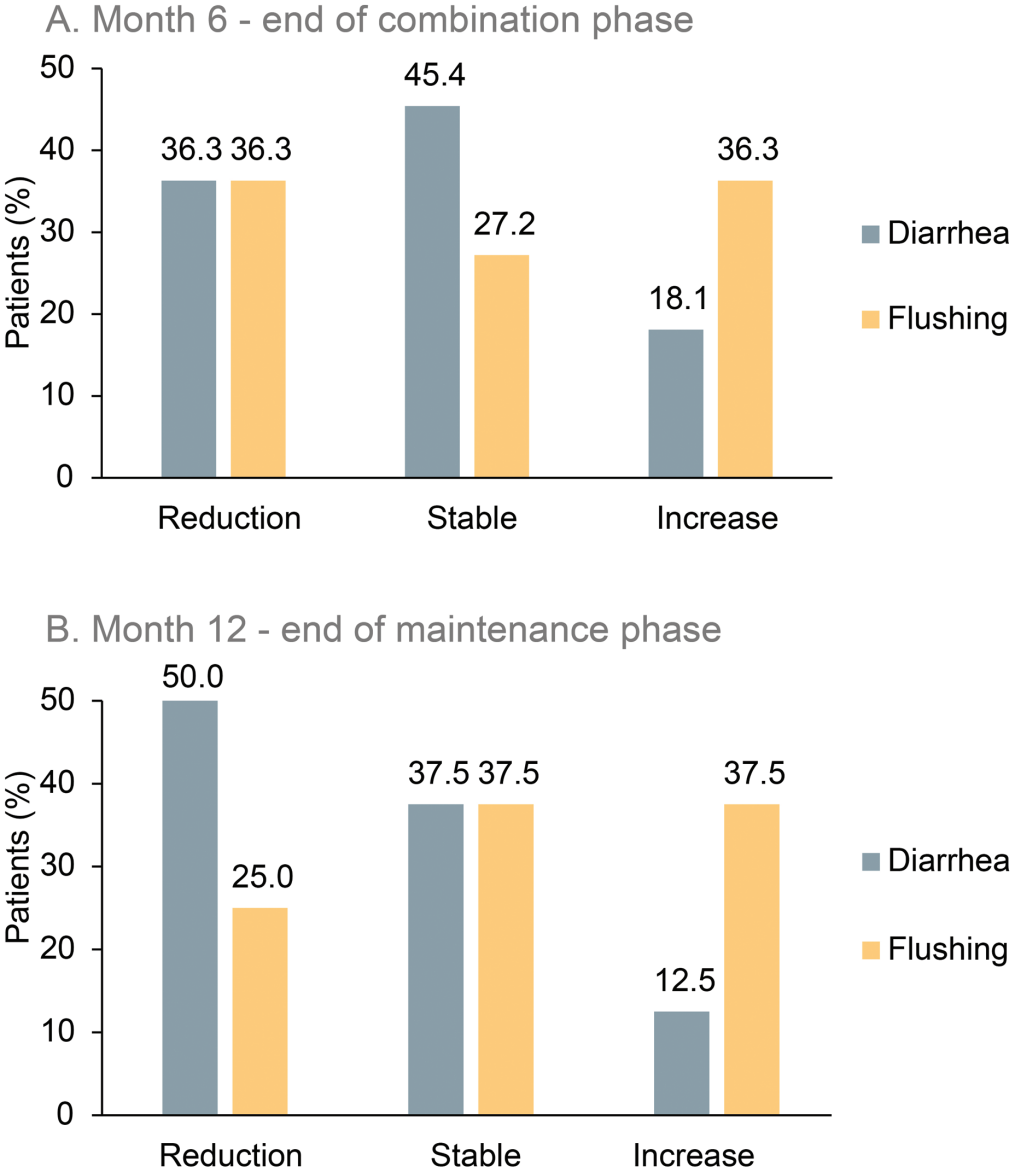
Symptomatic response in categories in terms of daily diarrhea and flushing episodes in patients with carcinoid syndrome due to serotonin-producing NET (**A**) at 6 months (end of combination phase) and (**B**) at 12 months (end of maintenance phase). Abbreviation: NET, neuroendocrine tumors.

In patients with F-NET, occurrence of diarrhea and flushing episodes appeared to be stable or decreased at 6 and 12 months (Fig. 4). No clear differences were observed either in the combination or in the maintenance phase for the patients’ QoL. During the combination phase, the mean EORTC QLQ-C30 global health status subscore tended to decrease from a mean of 67.2 (range, 25-100) at baseline to 63.1 (0-100) at month 6, as did the mean subscores of functional scales, representing a lower QoL and a lower level of functioning, respectively. The mean QLQ-C30 global health status subscore essentially remained unchanged between the end of the combination phase and the end of the maintenance phase in all study groups (not shown). Similarly, only slight or no changes were observed for the other QLQ-C30 and QLQ-GINET21 symptom subscores assessed (not shown).

### Adverse Events

Fifty-five from 57 (96.5%) patients reported treatment-emergent AEs (TEAE) in the combination and 33 from 37 (89.2%) in the maintenance phase (Table 3). TEAE mostly were NCI-CTCAE grade 1 or 2 (combination: 87.7%, maintenance: 89.2%). Forty-three (75.4%) patients presented with at least 1 TEAE considered related to treatment. Seventeen (29.8%) patients experienced at least 1 serious TEAE (SAE) during the combination phase and 11 (29.7%) during maintenance (Table 3). The most frequent TEAE by primary system organ class were gastrointestinal disorders, general disorders (fatigue), and blood and lymphatic system disorders. Most common grade 3/4 events in the combination phase included nausea (14.0%), thrombocytopenia (12.3%), and neutropenia (8.8%). The incidence of hematological TEAE was higher during the combination than during the maintenance phase. TEAE reported in ≥5% of the patients (all grades) are presented in Supplementary Table S2.

**Table 3.** Safety profile during the combination and maintenance phase.

Patients, *n* (%)	Combination phase	Maintenance phase		
		F-NET	NF-NET	
	LAN + TMZ (*n* = 57)	LAN (*n* = 11)	LAN (*n* = 14)	W&W (*n* = 12)
Any AE	56 (98.2)	9 (81.8)	13 (92.9)	11 (91.7)
Any TEAE	55 (96.5)	9 (81.8)	13 (92.9)	11 (91.7)
Any related TEAE	43 (75.4)	4 (36.4)	9 (64.3)	9 (75.0)
Any serious TEAE	17 (29.8)	3 (27.3)	4 (28.6)	4 (33.3)
Any related serious TEAE	4 (7.0)	0	0	0
TEAEs leading to withdrawal	9 (15.8)	0	1 (7.1)	0
Number of deaths	1 (1.8)^a^	1 (9.1)^b^	1 (7.1)^c^	1 (8.3)^c^

Death due to ^a^cardiac insufficiency, ^b^pneumonia, ^c^multiorgan failure due to cancer progression (all not considered related to study medication).

Abbreviations: AE, adverse event; F, functioning; LAN, lanreotide autogel; NET, neuroendocrine tumor; NF, nonfunctioning; TEAE, treatment-emergent adverse event; TMZ, temozolomide; W&W, watch and wait.

During the combination phase, 9 patients (15.8%) displayed at least 1 TEAE that led to withdrawal of study medication (24 events): bile duct stenosis (3 events); jaundice, tricuspid valve incompetence (2 events each); arthralgia, asthenia, blood bilirubin increase, cardiac failure, cholangitis, congestive cardiomyopathy, C-reactive protein increase, depression, diabetes mellitus, edema, hyperbilirubinemia, ileus, leucocytosis, myalgia, nausea, seronegative arthritis, and vomiting (1 event each). TEAE leading to discontinuation were serious except 5 events and not considered treatment-related except 4 events (bilirubin increase, jaundice, nausea, and vomiting) reported in 2 patients. During the maintenance phase, cholangitis resulted in the withdrawal of study medication in 1 patient from the LAN-treated NF-NET group.

During the study period, 4 deaths were reported resulting from SAE not considered related to study medication. During the combination phase, one patient with a history of diabetes and hypertension for over 10 years in addition to pneumonectomy due to lung carcinoma died of cardiac insufficiency. During maintenance, 3 patients died: one patient due to pneumonia in the group of LAN-treated patients with F-NET, and 2 patients due to multiorgan failure because of cancer progression (one in each group of patients with NF-NET).

No abnormalities in vital signs were observed throughout the study. Abdominal ultrasound exams were performed in 5 patients during the study. In one patient, new gallbladder stones and sludge were found during the maintenance phase.

## Discussion

This is the first study to investigate in a prospective design a combination of an SSTR-directed therapy and an alkylating agent in a mixed population of patients with advanced, progressive GEP-NET or GEP-NET of unknown origin.

Almost three-quarters of patients treated with LAN plus TMZ achieved disease control at 6 months of treatment, which was defined as either SD, PR, or CR. In most of these patients, SD (*n* = 33; 67.3%) was observed, while PR (*n* = 3; 6.1%) was a rare event, and no CR was observed. Around 25% had been nonresponders to the combination therapy of LAN plus TMZ. A further 10% of patients experienced disease progression during the observation period.

Administration of somatostatin analogs is an established treatment of patients with metastatic NET.^6^ Our study population consisted of mostly heavily pretreated patients, the vast majority of whom had previously received somatostatin analogs, and about half also had PRRT. Therefore, the patients included in this study likely presented with features of a more aggressive tumor biology and in urgent need of an effective yet tolerable follow-up therapy. In an analysis of 11 prospective trials of LAN monotherapy in advanced and/or progressive GEP-NET, PR rates generally ranged between 0% and 8% (except in one study 31%), SD rates between 15% and 89%, irrespective of prior disease progression status (reviewed by Sideris et al^37^). In the randomized CLARINET trial, 67 of 101 patients (66%) treated with LAN remained alive without progression within 12 months of treatment vs 49% of patients on placebo.^6^ However, patients with NF-NET with low proliferative activity (Ki-67 ≤10%) and mostly no disease progression at 3-6 months before start of LAN were included.

For treatment of neuroendocrine neoplasms (NEN), TMZ has been used in different combinations, most frequently with capecitabine. In a retrospective study, in patients with advanced pancreatic NET, who mostly had documented progression, capecitabine and TMZ (*n* = 100) were not superior compared with TMZ alone with respect to median PFS (*n* = 38). However, although not significant, a trend toward higher ORR was observed with the combination treatment than with TMZ alone (51% vs 34%; *P* = .088).^38^ Results of a retrospective chart review study (*n* = 116) showed encouraging PFS, overall survival, and ORR in patients with advanced NEN (progression status at inclusion not provided) with a DCR of 73% with capecitabine and TMZ,^39^ ie, comparable to our results. Most of the patients included had pancreatic (41%) or small intestinal NET (32%), 47% previously had received somatostatin analogs, 58% surgery, and 31% chemotherapy, similar to our patient population. According to a meta-analysis of 15 small studies—all retrospective but one—(*n* = 384) the DCR of capecitabine and TMZ was similar to our study (73%) and greater than with TMZ alone.^40^

In the recently reported prospective randomized phase II trial (*n* = 144), in patients with advanced pancreatic NET with documented disease progression within ≤12 months, the combination of capecitabine and TMZ resulted in a median PFS of 22.7 months, while patients treated with TMZ alone had a median PFS of 14.4 months (*P* = 0.023)^21^ ORR (34% and 40%, respectively, with mono- and combination therapy) did not differ between treatment arms. Approximately 50% of the patients in both treatment arms had concurrently received a somatostatin analog. More patients with grade 1 NET were included in the combination arm, what may have impacted the more favorable PFS with the combination therapy. The study is not directly comparable to our study since only 35% had a primary tumor in the pancreas, and the vast majority had their primary tumor at other sites of the digestive tract; this may also explain why ORR are lower in our study. More prospective data are available on the combination of LAN and TZM from the ATLANT study in thoracic carcinoids. In this 12-month prospective single-arm phase II study, 40 patients with progressive well-differentiated bronchial or thymic carcinoids were treated with combined LAN and TMZ.^41^ Locally assessed DCR at 9 months was 35.0%, median PFS 37.1 weeks, suggesting that LAN and TMZ could offer an effective treatment option in these patients.

However, it remains unclear that what is the best drug for combination when using TMZ to treat GEP-NET and selection may vary by type of tumor and treatment goal. When combining TMZ with bevacizumab in a phase II study in 34 patients with locally advanced or metastatic NET (56% carcinoid, 44% pancreatic NET), an ORR of 15% and a median PFS of 11.0 months were observed.^14^ With everolimus and TMZ, 40% of patients with advanced pancreatic G1/G2-NET of a prospective phase I/II study experienced PR, the median PFS being 15.4 months.^16^ However, this combination was associated with significant toxicity related to either everolimus (hyperglycaemia, mucositis, and pneumonitis) or TMZ (lymphopenia and thrombocytopenia). Treatment with thalidomide and TMZ in a mixed NET patient population was associated with biochemical and radiological response rates of 40% and 25%, respectively.^15^

Response to TMZ therapy may vary by site of origin and is best established in pancreatic NET. In our study, LAN and TMZ were effective in all subgroups analyzed. Differences in DCR indicating a greater clinical benefit for patients with a small intestinal NET location, for patients with lower hepatic tumor load (≤25%), and for patients with lower Ki-67 or WHO grade (Ki-67 index <3%; G1) probably indicate the prognostic significance of these parameters rather than a specific treatment benefit. However, some patients with pancreatic NET may have had higher benefit when considering that more patients with pancreatic NET achieved partial remission than patients with small intestinal NET; however, PFS was comparable between pancreatic and small intestinal NET. According to other studies, TMZ appeared to be more active in pancreatic vs other NET when combined with bevacizumab or thalidomide.^14,15^ Although patient numbers were low, our findings of the DCR in LAN-treated patients with NF-NET being numerically higher than in untreated patients suggest higher efficacy due to LAN maintenance therapy compared with a watch-and-wait strategy. The value of maintenance LAN therapy is strengthened by the results of the REMINET study indicating higher DCRs and more durable PFS when chemotherapy in duodeno-pancreatic NET was followed after 6 months by LAN maintenance therapy as compared to placebo.^42^ With respect to functional activity, more patients with F-NET reported improved or unchanged symptoms than worsening of symptoms, particularly of diarrhea, both at the end of the combination and maintenance phases. Together with the observed reduction of 5-HIAA levels, this suggests efficacy of LAN and TMZ also in patients with hormone-producing NET.

Our study is one of the few to report *MGMT* methylation as well as protein expression results in relation to TMZ treatment efficacy in a prospective design. We observed a trend toward *MGMT* promoter methylation to reflect response to TMZ treatment while a corresponding loss of MGMT expression could not be observed. Our results may confirm previous retrospective studies and one prospective study that indicated a putative predictive/prognostic role of *MGMT* promoter methylation status in NET.^21-23^ However, the 2 methods used to determine MGMT status (methylation vs expression) did not produce consistent results, and *MGMT* methylation may be more informative than MGMT expression which is more susceptible to influencing factors. In our study, only in approximately 60% of tumors with a methylated *MGMT* promoter, we observed concurrent loss of gene expression. Accordingly, contradictory results for MGMT promoter methylation and protein expression have been reported.^21^ MGMT promoter methylation and expression thus currently appear not to be suitable as predictive markers in NET with regard to response to TMZ treatment. Further comparative studies that include a control arm without TMZ are needed.

The advantage of combining LAN with TMZ is a lower toxicity as compared to other combinations. In general, treatment was well-tolerated, most of the patients experiencing grade 1 or 2 TEAE. The most frequently reported TEAE were consistent with a higher incidence particularly of gastrointestinal side effects known to be associated with LAN or TMZ treatment^8,43^ and hematologic side effects which are, among others, in particular, associated with TMZ.^43^ In 9 (15.8%) patients, TEAE resulted in medication withdrawal during the combination phase, of whom only 4 patients discontinued due to an AE related to a study drug. Similar withdrawal rates due to AE have been reported in trials investigating efficacy and safety of targeted therapies such as everolimus, eg, a withdrawal rate of 17% in patients with advanced pancreatic NET.^44^ Deaths during both treatment phases were considered unrelated to medication. Previous studies showed a similar acceptable toxicity profile of TMZ, allowing safe administration with other antitumor treatments in NEN.^14-20,40^

The main limitations of our study are the lack of a randomization into the combination vs monotherapy of TMZ treatment and the low number of patients during maintenance therapy in each of the subgroups, limiting the interpretation of the results. Therefore, we cannot demonstrate to what extent either lanreotide or TMZ contributed to the effect of combined treatment or whether this combination resulted in a synergistic effect. Moreover, the limited exposure to combination therapy of 6 months may have impacted the PFS results while in other studies TMZ-based therapy is given for up to 12 months. Patients were included in the trial based on tumor progression within 12 months, however, data on the time frame of progression were not available for the different primary sites. Finally, subgroup analyses resulted in small patient numbers and hence these data may only be interpreted as explorative. Especially, due to the low number of patients with unknown primary of less than 10% of the total population, we cannot draw any conclusions from the results obtained in this patient group. On the other hand, one of the strengths of the study is central assessment of imaging and of biochemical data minimizing potential observer and inter-test variabilities as well as assessment of MGMT status in a prospective design. Furthermore, this study provides prospective data on TMZ-based therapy in digestive nonpancreatic NET.

The findings of this phase II study show that treatment with LAN 120 mg/4 weeks and TMZ is feasible and a potential treatment option for patients with progressive F- or NF-NET irrespective of the primary site in the digestive tract. Clinical benefit should be confirmed in a future randomized clinical trial specifically designed to evaluate the role of TMZ in nonpancreatic NET, and further evaluating the role of maintenance therapy of LAN after chemotherapy.

## Supplementary Material

Supplementary material is available at *The Oncologist* online.

oyad325_suppl_Supplementary_Figures

oyad325_suppl_Supplementary_Tables

## Data Availability

Qualified researchers may request access to patient-level study data that underlie the results reported in this publication. Additional relevant study documents, including the clinical study report, study protocol with any amendments, annotated case report form, statistical analysis plan and dataset specifications may also be made available. Patient level data will be anonymized, and study documents will be redacted to protect the privacy of study participants. Where applicable, data from eligible studies are available 6 months after the studied medicine and indication have been approved in the US and EU or after the primary manuscript describing the results has been accepted for publication, whichever is later. Further details on Ipsen’s sharing criteria, eligible studies and process for sharing are available here (https://vivli.org/members/ourmembers/). Any requests should be submitted to www.vivli.org for assessment by an independent scientific review board.
