# Supplementary material for: Combined Lanreotide Autogel and Temozolomide Treatment of Progressive Pancreatic and Intestinal Neuroendocrine Tumors: The Phase II SONNET Study
Source: Oncologist. 2024 Jan 11;29(5):e643–54. doi: 10.1093/oncolo/oyad325 (PMC11067796; doi:10.1093/oncolo/oyad325)
Supplement: oyad325_suppl_Supplementary_Figures [file oyad325_suppl_supplementary_figures.docx]

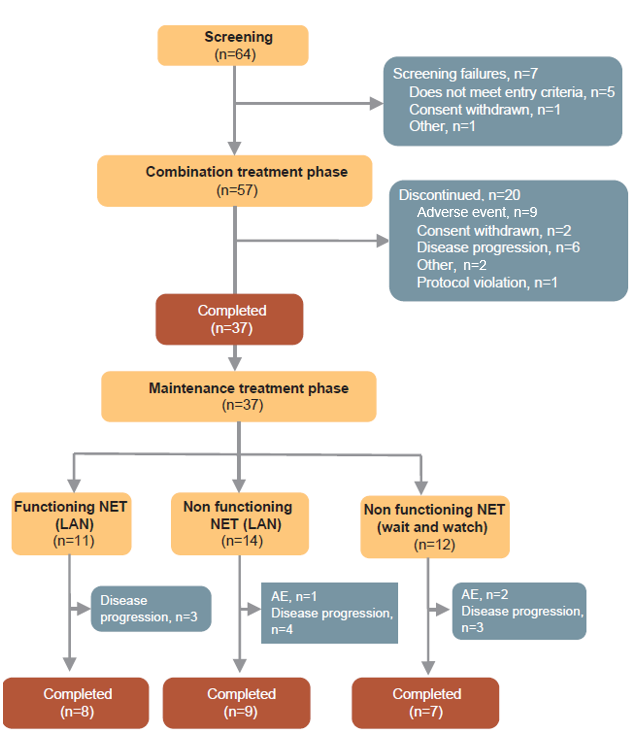


**Supplemental Figure 1.** Patient disposition. AE: adverse event, LAN: lanreotide autogel, NET: neuroendocrine tumor.


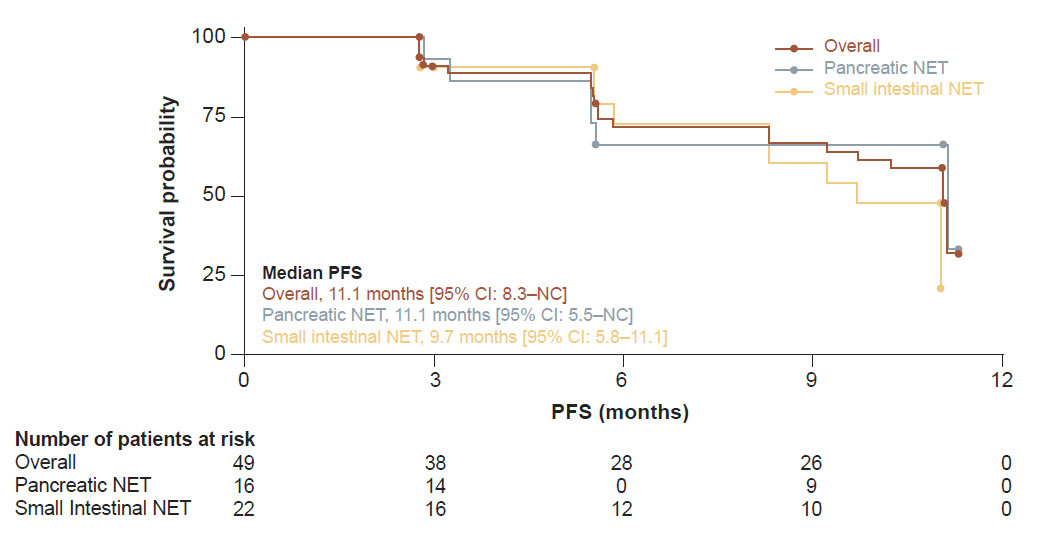


**Supplemental Figure 2.** Estimated median PFS overall and by pancreatic vs. small intestinal primary tumor location (ITT). CI: confidence interval, ITT: intention-to-treat, PFS: progression-free survival, NC: not calculated, NET: neuroendocrine tumor.
