# Supplementary material for: Combined Lanreotide Autogel and Temozolomide Treatment of Progressive Pancreatic and Intestinal Neuroendocrine Tumors: The Phase II SONNET Study
Source: Oncologist. 2024 Jan 11;29(5):e643–54. doi: 10.1093/oncolo/oyad325 (PMC11067796; doi:10.1093/oncolo/oyad325)
Supplement: oyad325_suppl_Supplementary_Tables [file oyad325_suppl_supplementary_tables.docx]

**Supplemental data**

**Supplemental Table 1.** Subgroup analysis of DCR after 6 months of combination treatment (ITT).

| **Domain** | **Variable** | **n** | **DCR** | |
| --- | --- | --- | --- | --- |
|  |  |  | **n (%)** | **[95% CI]** |
| Functional status | F-NET | 17 | 12 (70.6) | [44.0–89.7] |
|  | NF-NET | 32 | 24 (75.0) | [56.6–88.5] |
| NET location | Pancreatic | 16 | 10 (62.5) | [35.4–84.8] |
|  | Small intestinal | 22 | 17 (77.3) | [54.6–92.2] |
|  | Other | 11 | 9 (81.8) | [48.2–97.7] |
| Grade | Grade 1 | 8 | 7 (87.5) | [47.3–99.7] |
|  | Grade 2 | 41 | 29 (70.7) | [54.5–83.9] |
| Hepatic tumor load | ≤25% | 41 | 31 (75.6) | [59.7–87.6] |
|  | >25% | 8 | 5 (62.5) | [24.5–91.5] |
| Ki-67 proliferation index | <5% | 13 | 11 (84.6) | [54.6–98.1] |
|  | 5–10% | 23 | 15 (65.2) | [42.7–83.6] |
|  | >10–20% | 13 | 10 (76.9) | [46.2–95.0] |

DCR: disease control rate, F: functioning, ITT: intention-to-treat, NET: neuroendocrine tumor, NF: non-functioning.

**Supplemental Table 2.** TEAEs (all grades) in ≥5%of the patients.

| **TEAEs (preferred term), n (%)** | **Combination phase**  **(n = 57)** | | **Maintenance phase**  **(n = 37)** | |
| --- | --- | --- | --- | --- |
|  | **All grades** | **Grade ≥3** | **All grades** | **Grade ≥3** |
| Nausea | 24 (42.1) | 8 (14.0) | 6 (16.2) | 0 (0.0) |
| Diarrhea | 21 (36.8) | 1 (1.8) | 5 (13.5) | 1 (2.7) |
| Vomiting | 19 (33.3) | 1 (1.8) | 4 (10.8) | 0 (0.0) |
| Fatigue | 19 (33.3) | 3 (5.3) | 11 (29.7) | 3 (8.1) |
| Thrombocytopenia | 16 (28.1) | 7 (12.3) | 4 (10.8) | 0 (0.0) |
| Abdominal pain | 12 (21.1) | 1 (1.8) | 8 (21.6) | 1 (2.7) |
| Constipation | 11 (19.3) | 0 (0.0) | 2 (5.4) | 0 (0.0) |
| Flatulence | 10 (17.5) | 0 (0.0) | 4 (10.8) | 0 (0.0) |
| Lymphopenia | 8 (14.0) | 0 (0.0) | 6 (16.2) | 1 (2.7) |
| Weight decreased | 8 (14.0) | 0 (0.0) | 5 (13.5) | 0 (0.0) |
| Anemia | 7 (12.3) | 1 (1.8) | 3 (8.1) | 0 (0.0) |
| Headache | 7 (12.3) | 0 (0.0) | 1 (2.7) | 0 (0.0) |
| Nasopharyngitis | 7 (12.3) | 0 (0.0) | 8 (21.6) | 0 (0.0) |
| Leukopenia | 6 (10.5) | 0 (0.0) | 2 (5.4) | 0 (0.0) |
| Neutropenia | 6 (10.5) | 5 (8.8) | 2 (5.4) | 1 (2.7) |
| Arthralgia | 6 (10.5) | 2 (3.5) | 4 (10.8) | 0 (0.0) |
| GGT increased | 6 (10.5) | 4 (7.0) | 3 (8.1) | 2 (5.4) |
| Rash | 5 (8.8) | 0 (0.0) | 5 (13.5) | 2 (5.4) |
| Hypertension | 5 (8.8) | 0 (0.0) | 5 (13.5) | 0 (0.0) |
| Abdominal distension | 4 (7.0) | 1 (1.8) | 1 (2.7) | 0 (0.0) |
| Asthenia | 4 (7.0) | 1 (1.8) | 4 (10.8) | 2 (5.4) |
| Peripheral edema | 4 (7.0) | 1 (1.8) | 3 (8.1) | 0 (0.0) |
| Pyrexia | 4 (7.0) | 0 (0.0) | 7 (18.9) | 0 (0.0) |
| Serum creatinine increased | 4 (7.0) | 0 (0.0) | 2 (5.4) | 0 (0.0) |
| Upper abdominal pain | 3 (5.3) | 0 (0.0) | 0 (0.0) | 0 (0.0) |
| Decreased appetite | 3 (5.3) | 0 (0.0) | 1 (2.7) | 0 (0.0) |
| Hypokalemia | 3 (5.3) | 0 (0.0) | 4 (10.8) | 0 (0.0) |
| Dizziness | 3 (5.3) | 0 (0.0) | 2 (5.4) | 0 (0.0) |
| Pain in extremity | 3 (5.3) | 0 (0.0) | 2 (5.4) | 0 (0.0) |
| Hematoma | 3 (5.3) | 0 (0.0) | 1 (2.7) | 0 (0.0) |
| Hepatic pain | 3 (5.3) | 0 (0.0) | 3 (8.1) | 0 (0.0) |
| Jaundice | 3 (5.3) | 0 (0.0) | 1 (2.7) | 1 (2.7) |
| Insomnia | 3 (5.3) | 0 (0.0) | 2 (5.4) | 0 (0.0) |
| Dyspnea | 3 (5.3) | 0 (0.0) | 3 (8.1) | 0 (0.0) |
| Vertigo | 3 (5.3) | 0 (0.0) | 1 (2.7) | 0 (0.0) |
| Serum bilirubin increased | 3 (5.3) | 2 (3.5) | 0 (0.0) | 0 (0.0) |

GGT: gamma-glutamyl transferase, TEAE: treatment-emergent adverse event
